# Examestane in advanced or recurrent endometrial carcinoma: a prospective phase II study by the Nordic Society of Gynecologic Oncology (NSGO)

**DOI:** 10.1186/1471-2407-14-68

**Published:** 2014-02-05

**Authors:** Kristina Lindemann, Susanne Malander, Rene D Christensen, Mansoor R Mirza, Gunnar B Kristensen, Elisabeth Aavall-Lundqvist, Ignace Vergote, Per Rosenberg, Karin Boman, Britta Nordstrøm

**Affiliations:** 1 Department of Gynecologic Oncology, Norwegian Radium Hospital, Oslo University Hospital, Nydalen 0424, PB 4953, Oslo, Norway; 2 Department of gynecologic oncology, Lund University Hospital, Lund, Sweden; 3 Research Unit of General Practice, Institute of Public Health, University of Southern Denmark, Odense, Denmark; 4 Department of Oncology, Rigshospitalet, Copenhagen University Hospital, Copenhagen, Denmark; 5 Department of Gynecologic Oncology and Institute for Medical Informatics, Norwegian Radium Hospital, Oslo University Hospital, Oslo, Norway; 6 Department of Oncology, Karolinska University Hospital, Stockholm, Sweden; 7 Department of Gynecologic Oncology and Leuven Cancer Institute, University Hospital Leuven, Leuven, European Union; 8 Department of Gynecologic Oncology, University Hospital, Linkjoeping, Sweden; 9 Department of Oncology, University Hospital, Umeaa, Sweden; 10 Department of Oncology, Karolinska University Hospital, Stockholm, Sweden

**Keywords:** Aromatase inhibitor, Exemestane, Endometrial cancer, Treatment, Phase II study

## Abstract

**Background:**

We evaluated the efficacy and safety of the aromatase inhibitor exemestane in patients with advanced, persistent or recurrent endometrial carcinoma.

**Methods:**

We performed an open-label one-arm, two-stage, phase II study of 25 mg of oral exemestane in 51 patients with advanced (FIGO stage III-IV) or relapsed endometrioid endometrial cancer. Patients were stratified into subsets of estrogen receptor (ER) positive and ER negative patients.

**Results:**

Recruitment to the ER negative group was stopped prematurely after 12 patients due to slow accrual. In the ER positive patients, we observed an overall response rate of 10%, and a lack of progression after 6 months in 35% of the patients. No responses were registered in the ER negative patients, and all had progressive disease within 6 months. For the total group of patients, the median progression free survival (PFS) was 3.1 months (95% CI: 2.0-4.1). In the ER positive patients the median PFS was 3.8 months (95% CI: 0.7-6.9) and in the ER negative patients it was 2.6 months (95% CI: 2.1-3-1). In the ER positive patients the median overall survival (OS) time was 13.3 months (95% CI: 7.7-18.9), in the ER negative patients the corresponding numbers were 6.1 months (95% CI: 4.1-8.2). Treatment with exemestane was well tolerated.

**Conclusion:**

Treatment of estrogen positive advanced or recurrent endometrial cancer with exemestane, an aromatase inhibitor, resulted in a response rate of 10% and lack of progression after 6 months in 35% of the patients.

**Trial registration:**

Trial identification number (Clinical Trials.gov): NCT01965080.

Nordic Society of Gynecological Oncology: NSGO–EC–0302.

EudraCT number: 2004-001103-35.

## Background

Endometrial cancer is the most common cancer of the female genital tract in many Western countries, and some of the highest incidence rates have been observed in European populations [1]. Long-term predictions imply that the burden of endometrial cancer will continue to increase in the forthcoming decades owing not only to the aging population but also to the obesity epidemic [2]. The majority of patients are diagnosed at an early stage with favourable prognosis. However, a considerable proportion of patients continue to present with locally advanced or recurrent disease.

There are two distinct endometrial tumour types, type I and type II endometrial cancer. They differ in pathogenesis, histology and prognosis. Type I tumours are often well differentiated and characterized by endometrioid histology. These tumours are hormone dependent but clinical data on the expression of hormone receptors is scarce. A recent cohort study reported estrogen receptor expression in two-third of the tumors [3]. Hormone therapy is a particularly attractive option for the treatment of advanced endometrial cancer because it is well tolerated and lacks the usual toxicities associated with chemotherapy. Accumulated experience with a variety of hormonal regimens suggests that between 15 and 30 percent of women respond to hormone therapy [4], with a correlation between receptor status and response to hormone therapy [5,6]. However, metastatic or recurrent disease may be different from primary disease as regards tumour biology and receptor status [7], and a recent Cochrane review found no evidence that hormonal treatment in any form improves survival in this patient group [8].

In postmenopausal women or after oophorectomy, the major source of circulating estrogen is conversion of adrenal androstenedione to estron by aromatase activity in adipose tissue with further conversion to estradiol [9]. In the first line treatment of postmenopausal women with hormone receptor-positive breast cancer, aromatase inhibitors (AI’s) have shown superior efficacy as compared with tamoxifen. However, there is very little clinical evidence on the efficacy of AI’s in endometrial cancer. Previous studies of the AI’s letrozole and anastrozole have only shown limited effect [10,11]. Exemestane is an oral irreversible steroidal AI and has been shown to decrease circulating estrogens in postmenopausal women [12]. Exemestane is given as an oral dose once daily and may therefore be a convenient drug in the treatment of advanced endometrial cancer. We present the final results of an open-label phase II study on the efficacy and safety of the aromatase inhibitor exemestane in the treatment of patients with advanced, persistent or recurrent endometrial carcinoma of endometrioid type. Preliminary results were previously presented at the 2006 Annual Meeting of the American Society of Clinical Oncology [13].

## Methods

### Study design

This open-label phase II study was designed to evaluate the efficacy and safety of exemestane in the treatment of patients with advanced, persistent or recurrent endometrial carcinoma of endometrioid type.

The study was designed and carried out in accordance with good clinical practice, the declaration of Helsinki and national laws. The local ethics committee at each participating center approved the study (Belgia: Commissie Medische Ethiek van Universitaire Ziekenhuizen KU Leuven; Danmark: Den Videnskabsetiske komite or Vejle og Fyns Amter; Norway: Regional Committees For Medical and Health Research Studies; Sweden: Regional ethical review board). All patients gave their written informed consent before study entry.

Patients received 25mg of oral exemestane once daily, preferable after a meal, on an outpatient basis.

### Eligibility criteria, randomization, and quality assurance

Patients with histologically confirmed advanced (FIGO stage III-IV) or relapsed endometrial cancer of endometrioid type not considered for curative treatment were eligible. Patients could have been treated by surgery, radiotherapy and/or chemotherapy. All patients had to have at least one measurable lesion located outside the previously irradiated area. Lesions located within irradiated area were considered as non-target lesions. Relapse was verified by cytology or histology. Time from any cancer treatment had to be at least 1 month. Patients were included irrespective of hormone receptor status. Only postmenopausal women with a WHO performance status of 0-2 were included. Adequate renal and hepatic function, defined as follows, was required: ASAT or ALAT of no more than 4 x upper normal limit (UNL) and serum creatinine <150 mmol/L. Exclusion criteria were: Patients with symptomatic brain metastases, a history of other primary malignancies except for carcinoma in situ of the cervix and basal cell carcinoma of the skin, a history of thromboembolic disease, congestive heart failure (NYHA classification >2) or any treatment that might interact with the study drug (i.e., carbamazine or cyclosporine).

The Nordic Society of Gynecologic Oncology (NSGO) data center checked all of the data collected on case report forms for consistency.

### Pathological review and immunohistochemical staining

Pathological review of the tumors was conducted on an institutional basis by pathologists well trained in gynecological pathology. Estrogen receptor (ER) staining was considered positive if nuclear staining in more than 10% of the tumor cells was 2+ or higher.

### Evaluations and follow-up

During the treatment period patients were seen every three months during the first year, every six months during the second year and thereafter until disease progression. Each clinical visit included laboratory tests (full blood cell count, serum creatinine, ASAT/ALAT), a physical examination, assessment of performance status and adverse events and tumor evaluation with computed tomography. Response was defined according to the RECIST criteria [14]. For evaluation of response rate, patients had to have at least one tumor assessment performed after start of therapy. For categorization as stable disease the disease had to have been stable for at least 6 months. Adverse events and toxicity were graded by the study investigators according to the National Institute Common Toxicity Scale version 2.0 [15]. Toxicities were recorded continuously and evaluated using the worst score over the whole treatment period for each patient.

### Statistical analyses

The primary endpoint measure was objective response rate. Secondary endpoints were toxicity, progression free (PFS) and overall (OS) survival. The response rate and survival endpoints were evaluated in each of two separate subsets: A) ER positive and B) ER negative tumors. In each subset the SWOG 2 stage design [16] was used with a targeted response rate of 30% and a rate of only 10% as unacceptable low. An accrual of 20 in the first stage (stage 1) and 15 in the second stage (stage 2) for each subset was targeted, resulting in approximate significance level and power of 0.05 and 0.9 respectively. In case of less than 2 responses observed in the first stage of the study, the respective subset would be closed. With 2 or more responses, the subset would continue to the second stage.

Objective response rate was evaluated based on the total number of evaluable patients in the subset. Progression free survival was defined as the time from study entry to disease progression or death of any cause. Patients still alive with no progression were censored at the date of their last follow-up visit. Overall survival was defined as the time from study entry to death of any cause. Patients still alive were censored at the date of their last follow-up visit. Survival was analyzed using the Kaplan-Meier method. Efficacy analyses were performed on all randomly assigned patients on an intention-to-treat basis. All patients were evaluable for safety analysis from the time of their first dose of treatment. The STRATA statistical package, version 10.0, was used for the analysis.

## Results

### Patients and follow-up

**Figure 1:**
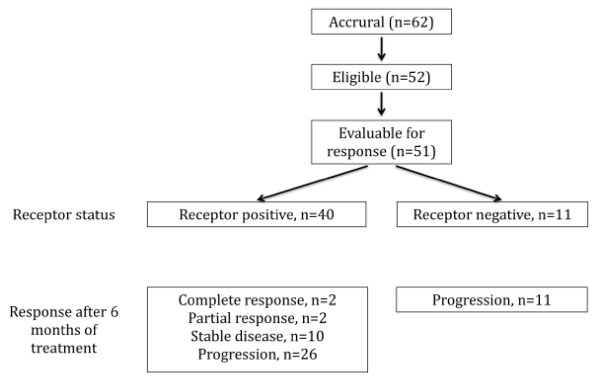
Enrollment, randomization and treatment response.

Between 01.03.2004 and 30.11.2006, 62 patients were screened for this trail, of which 52 fulfilled all eligibility criteria and were enrolled (Figure 1). After accrual of 12 patients with ER negative tumors who all progressed shortly, investigators stopped recruitment to this group. Thirty-four (65.4%) patients with relapse were included. Ten patients suffered from relapse after surgery alone, 8 of whom presented with disease outside the pelvis. Disease was primarily metastatic in 18 (34.6%) patients. Description of treatment given prior to inclusion in the study is given in Table 1. All patients but four had undergone surgery with hysterectomy and bilateral salpingo-oophorectomy. These four patients with primarily metastatic disease had a diagnostic curettage before treatment with radiation and/or chemotherapy.

**Table 1:** Baseline patient characteristics

**Characteristic**	**ER positive**		**ER negative**		**Total**	
	**No. of patients**	**%**	**No. of patients**	**%**	**No. of patients**	**%**
**No. of patients**	40	76.9	12	23.1	52	100
**Age, years**						
Range	42.9–82.8		59.4–82.5		42.9–82.8	
Median	70.0		67.7		69.5	
FIGO stage*					**52**	**100**
I	13	32.5	9	75.0	22	42.3
II	6	15.0	1	8.3	7	13–5
III	11	27.5	2	16.7	13	25.0
IV	10	25.0	0	0	10	19.2
**Grade**					**52**	**100**
Well differentiated	7	17.5	0	0	7	13.5
Mod. differentiated	17	42.5	7	58.3	24	46.2
Poorly differentiated	14	35.0	5	41.7	19	36.5
Not specified	2	5.0	0	0	2	3.8
**Disease status at inclusion**					**52**	**100**
Relapse	26	65	8	66.7	34	65.4
Primary metastatic disease	14	35	4	33.3	18	34.6
**Previous treatment**					**52**	**100**
Surgery alone	11	27.5	1	8.3	12	23.1
Surgery and radiation	8	20.0	1	8.3	9	17.3
Surgery and chemotherapy	7	17.5	3	25.0	10	19.2
Surgery, radiation and chemotherapy	14	35.0	7	58.3	21	40.4

*Stage at primary diagnosis.

### Treatment compliance and toxicity

Exemestane was well tolerated in this study. The reported incidence of non-hematological toxicity is shown in Table 2. Most of the events were of grade 1 or 2. Grade 3-4 anorexia was reported by 2 patients (3.8%). There were 3 cases (5.8%) of venous thrombosis. The reported hematological toxicity is shown in Table 3. Grade 3-4 anemia was reported in 28 (53.8%) of the patients.

**Table 2:** Non-hematologic toxicity by toxicity grade (NCI-CTC)

**Toxicity**	**1**		**2**		**3**		**4**	
	**No.**	**%**	**No.**	**%**	**No.**	**%**	**No.**	**%**
Anorexia	8	15.4	5	9.6	1	1.9	1	1.9
Nausea	11	21.2	2	3.8	1	1.9	0	0
Vomiting	5	9.6	1	1.9	1	1.9	0	0
Abdominal pain	4	7.7	4	7.7	1	1.9	0	0
Hot flushes	6	11.5	3	5.8	0	0	0	0
Sweating	7	13.5	4	7.7	0	0	0	0
Fatigue	9	17.3	9	17.3	2	3.8	0	0
Dizziness	5	9.6	1	1.9	1	1.9	0	0
Headache	4	7.7	2	3.8	0	0	0	0
Weight gain	4	7.7	1	1.9	0	0	0	0
Insomnia	5	9.6	4	7.7	0	0	0	0
Edema	3	5.8	1	1.9	0	0	0	0
Venous thrombosis	0	0	0	0	3	5.8	0	0

**Table 3:** Hematologic toxicity by toxicity grade (NCI-CTC)

**Toxicity**	**1**		**2**		**3**		**4**	
	**No.**	**%**	**No.**	**%**	**No.**	**%**	**No.**	**%**
WBC/granulocytes	2	3.8	0	0	0	0	0	0
Platelets	0	0	0	0	1	1.9	0	0
Hemoglobin	7	13.5	2	3.8	1	1.9	27	51.9

### Tumor response and survival

One patient with ER negative tumor stopped treatment after 3 weeks owing to deteriorated performance status and clinical signs of progression but without radiologic evaluation. Objective evaluation of response according to RECIST was done in 51 patients. During treatment and follow-up, a total of 43 patients had disease progression (PD), while 8 patients had no progression. One patient died from intercurrent disease (intracranial bleeding), while in complete remission (CR) for 23 months. At the end of follow-up one patient was in CR with PFS of 55.3 months, 2 had partial remission (PR) with PFS of 26.6 and 25.6 months, respectively. Four patients had stable disease (SD) with PFS of 40.7, 9.6, 7.9 and 10.9 months, respectively. Twelve patients with ER negative tumors were registered. In the 11 ER negative patients evaluable for response, no response was observed. The trial was stopped prematurely in the ER negative group due to lack of recruitment. In the ER positive group the trial advanced into stage 2 and a total of 40 patients were recruited. The response status after 6 months of treatment was: Progressive disease in 26 patients, complete response in 2, partial response in 2 and stable disease in 10 patients. This gives an overall response rate of 10% and absence of progression in 14 patients (35%).

**Figure 2:**
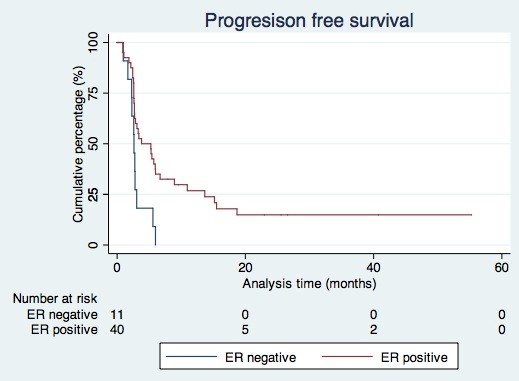
Progression free survival according to receptor status.

For the total group of patients, the median progression free survival was 3.1 months (95% CI: 2.0-4.1). In the group of ER positive patients the median progression free survival was 3.8 months (95% CI: 0.7-6.9). Progression free survival was 2.6 months (95% CI: 2.1-3.1) in the group of ER negative patients (Figure 2). At the end of the study, 7 patients in the ER positive group were progression free.

**Figure 3:**
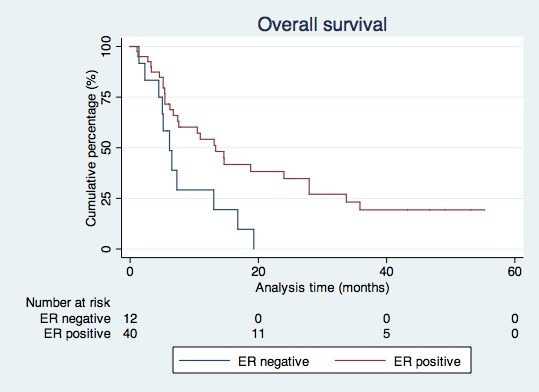
Overall survival according to receptor status.

During follow-up 38 patients (73.1%) died. For the total group of patients, the median overall survival time was 10.9 months (95% CI: 3.4-18.0). In the group of ER positive patients the median overall survival time was 13.3 months (95% CI: 7.8-18.9). ER negative patients survived for a median of 6.1 months (95% CI: 4.1-8.2) (Figure 3).

## Discussion

The treatment with exemestane was generally well toletated. The response rate in the ER positive group was 10%. In the design of the study, a response rate of 10% or less was considered as of no clinical interest. In retrospect, the use of response rate as endpoint for this study can be questioned. Long term stabilization, such as lack of progression at 6 months may be a more relevant endpoint. We observed a lack of progression in 35% of patients at 6 months, which seems of clinical interest. Tumors are heterogeneous, and the definition used for ER positivity in this study was 2+ or more in staining intensity for at least 10% of tumor cells. This means that a substantial proportion of the tumor cells may have stained negative for ER in a number of tumors included in the ER positive group. The tumor cells that stained negative for ER in the ER positive patient group may not have responded to the treatment, and the progressions in this group may be attributable to these tumor cells. Patients with long-term response or stabilization of the disease may show more uniform ER positivity of the tumor cells but unfortunately the degree of staining was not specified in the study. We were therefore not able to validate this hypothesis.

Recruitment in the ER negative subset was stopped prematurely after recruitment of 12 patients because of lack of response and rapid progression in these patients. We cannot conclude too strongly about this subset due to the small number of patients, but do consider the results as disappointing.

There are other weaknesses in this study. Pathological review and histological staining of the tumors were performed on an institutional basis. However, all institutions were university hospitals with well-trained gynecological pathologists. Immunostaining of estrogen receptor is well established, and there is broad consensus about evaluation of staining results. In 24 cases (46.1%), receptor status was evaluated in the primary tumor only. However, expression of hormone receptors may change during progression of the disease and receptor status of metastatic disease may not always correspond to the receptor expression of the primary tumor. The trial was designed as a single-arm phase II study and hence did not include a control arm. Therefore we cannot exclude the lower progression rate observed in the ER positive group being attributable to a difference in tumor biology not related to the ER receptor status.

Two previous studies of the aromatase inhibitors letrozole and anastrozole in endometrial cancer patients have reported disappointing results. Ma et al. reported a response rate of 9% in 32 patients in abstract form [10]. There was no significant correlation with hormone receptor status, but data on receptor status was available in only one third of the patients. Another phase II trial of anastrozole observed only two partial responses in 23 patients. They reported ER and PR positivity in 21.7% of the patients and patients enrolled in the study were older and had mainly non-endometrioid tumors [11]. In a recent abstract report on 42 patients with recurrent endometrial cancer, the effects of concomitant letrozole and the mTOR inhibitor everolimus were studied [16]. The response rate was 20% and the 12 month progression-free rate was 37%. The previously reported low response rates of endocrine therapy in endometrial cancer, especially with AI’s, may reflect the need to identify the subset of women most likely to respond to such therapy. In our study we therefore selected type I endometrial cancers, and 77% of the tumors in our study population were ER positive. In this subgroup, we report a response rate of 10%. The lack of progression within 6 months in 35% of the patients is of clinical interest and may warrant further evaluation in this group of patients. In receptor negative patient, no responses were observed, and patients progressed quickly. In this subgroup, further evaluation of this kind of treatment seems of less interest. In order to evaluate the potential benefit of endocrine treatment, hormone receptor status should always be evaluated, preferably in the metastatic sites, but at least in the primary tumor.

## Conclusions

Treatment of estrogen positive advanced or recurrent endometrial cancer with exemestane, an aromatase inhibitor, resulted in a response rate of 10% and a lack of progression at 6 months of treatment in 35% of patients with ER positive tumors. The treatment was well tolerated.

## Competing interests

The authors declare that they have no competing interests.

## Authors’ contributions

KL: Performed the statistical analysis, interpretation of data and drafted the manuscript. SM: Acquisition of data. RDC: Helped with performing the statistical analyses, interpretation of data and helped to draft the manuscript. MRM: Participated in designing the study and data acquisition. Helped to draft the manuscript. GBK: Participated in trial design. Coordinated the study. Data acquisition and interpretation and helped to draft the manuscript. EA-L: Participated in study design, acquisition of data and interpretation of data. Helped to draft the manuscript. IV: Acquisition of data. PR: Acquisition of data. KB: Acquisition of data. BN: Acquisition of data. All authors read and approved the final manuscript. All authors agreed to be accountable for all aspects of the work in ensuring that questions related to the accuracy or integrity of any part of the work are appropriately investigated and resolved.

## Pre-publication history

The pre-publication history for this paper can be accessed here:

http://www.biomedcentral.com/1471-2407/14/68/prepub

## List of investigators

The following investigators are acknowledged for their contribution to the study:

Norway

Oslo: H. Oksefjell, J. Kaern, G. Kristensen. Tromsoe: A.B. Vereide

Sweden

Linkoeping: P. Rosenberg. Lund: S. Malander, Bergenfelt, M. Kalling. Stockholm: B. Nordstrom. Orebro: M. Graflund. Umea: H Persson

Denmark

Odense: M. Mirza. Aalborg: B.Lund

Belgia:

Leuven: I.Vergote. Antwerp: A.Makar
